# Supplementary material for: Loss of NDR1/2 kinases impairs endomembrane trafficking and autophagy leading to neurodegeneration
Source: Life Sci Alliance. 2022 Nov 29;6(2):e202201712. doi: 10.26508/lsa.202201712 (PMC9711861; doi:10.26508/lsa.202201712)
Supplement: Supplementary file 1 [file LSA-2022-01712_SdataF1_F2_F3_F4_F5_FS1_FS2_FS3_FS4.pdf]

Fig 1F

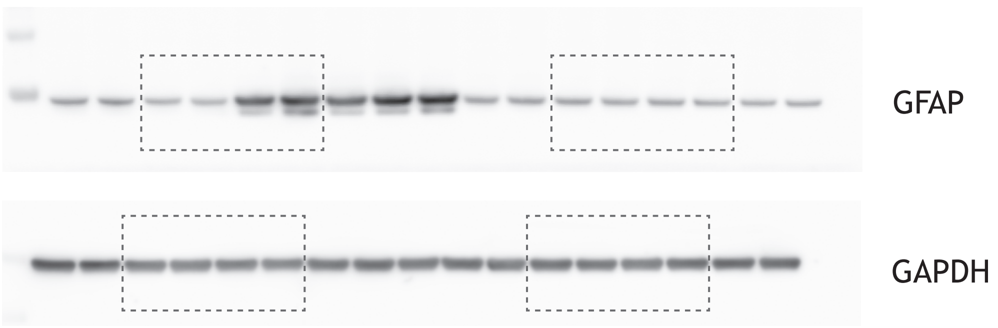

Fig 2G

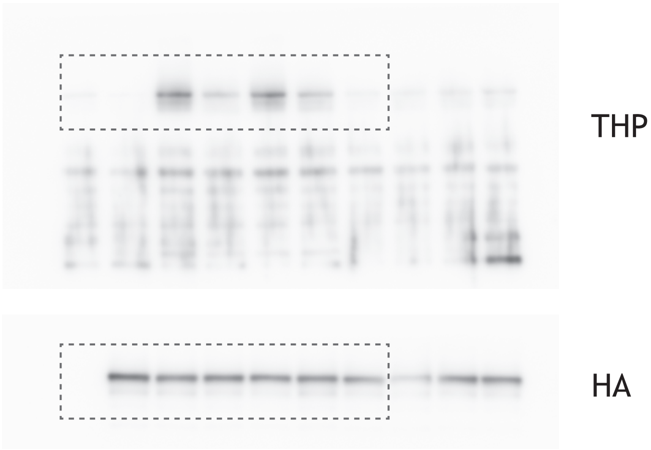

Fig 3B

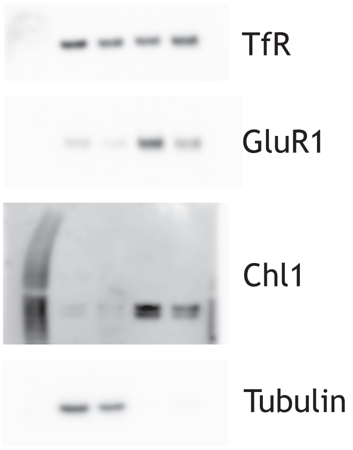

Fig 3E

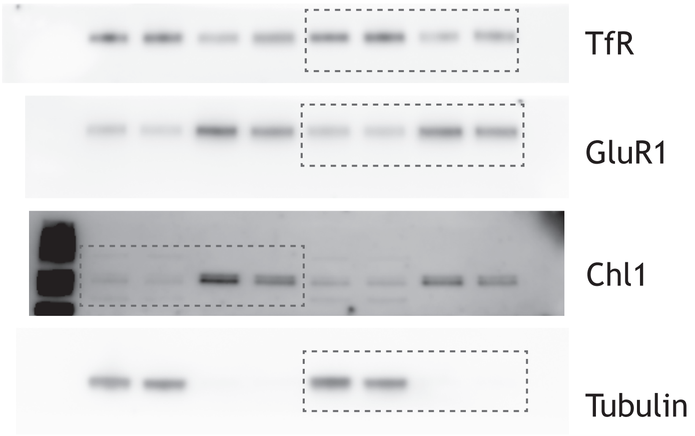

Fig 4B

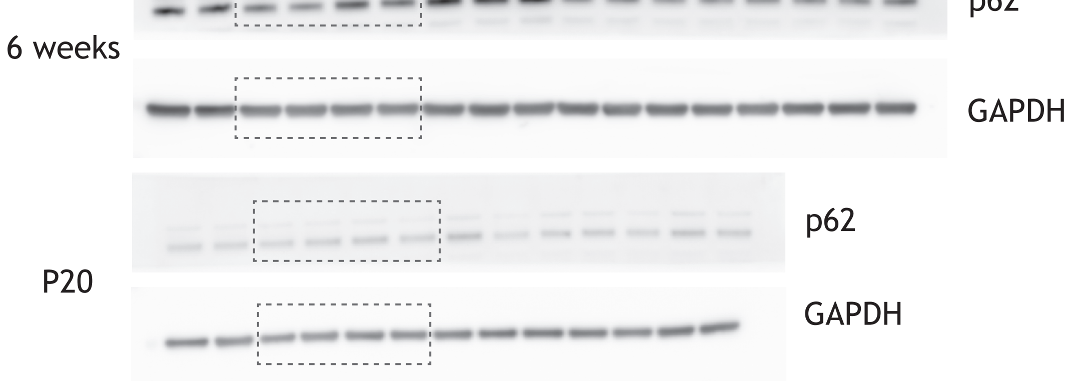

Fig 4F

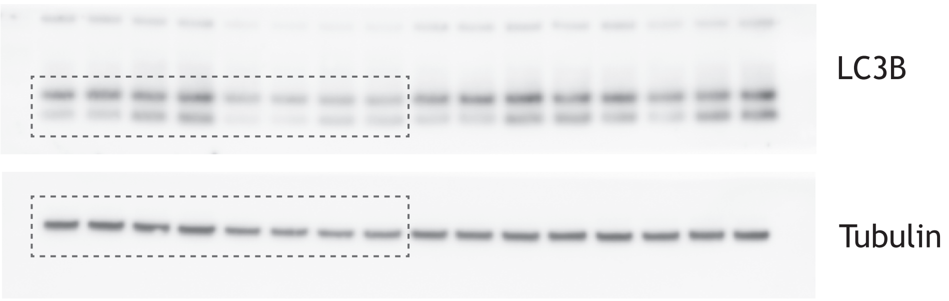

Fig 4G

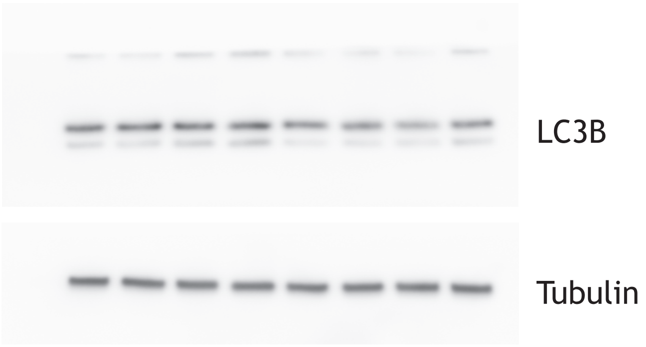

Fig 5B

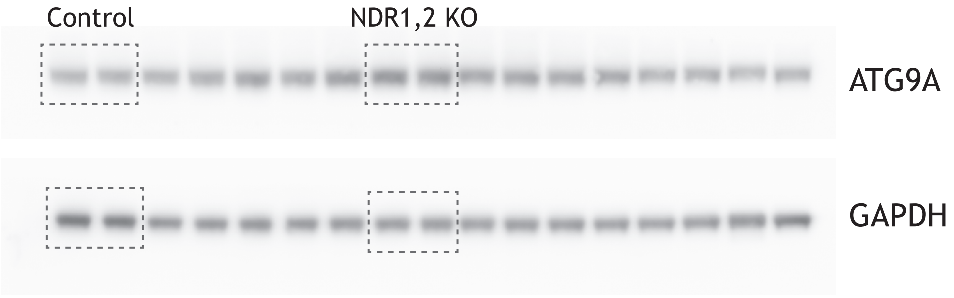

Fig 5F

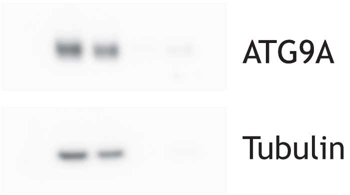

Fig 5G

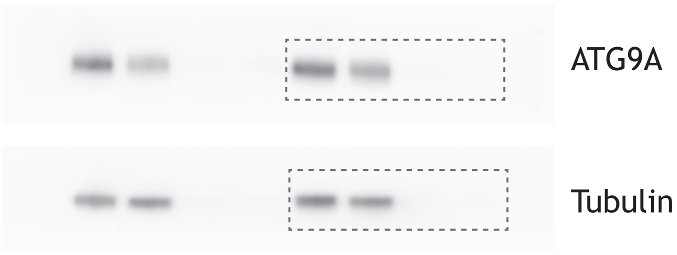

Fig S1C

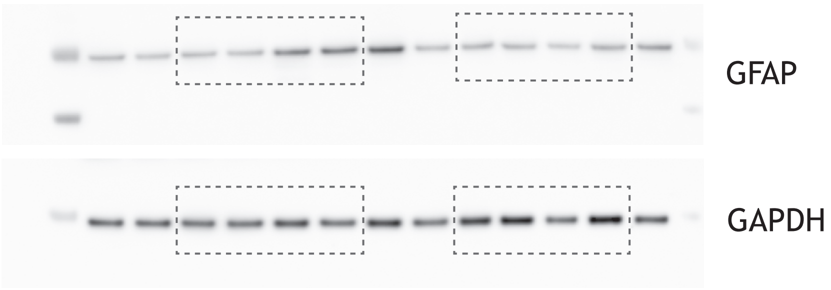

Fig S1F

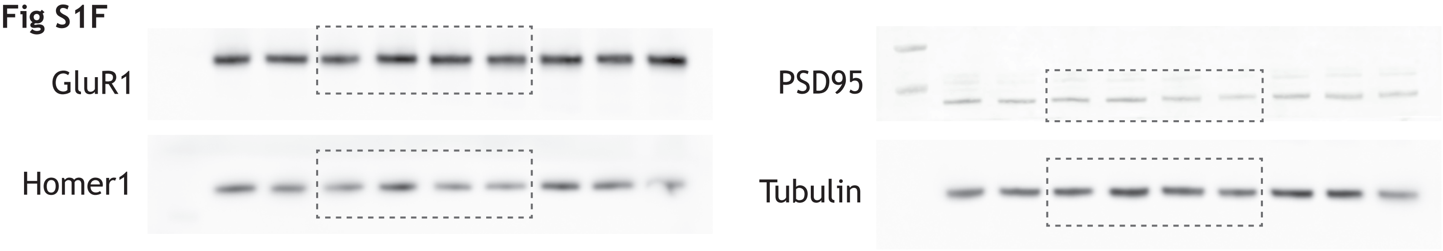

Fig S2F

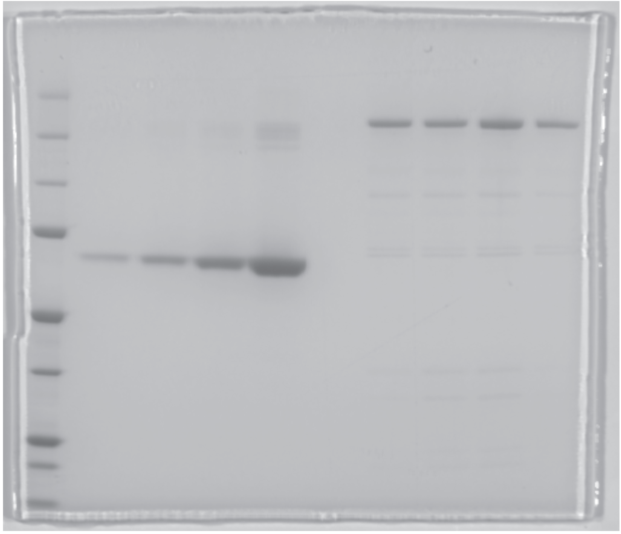

Fig S2G

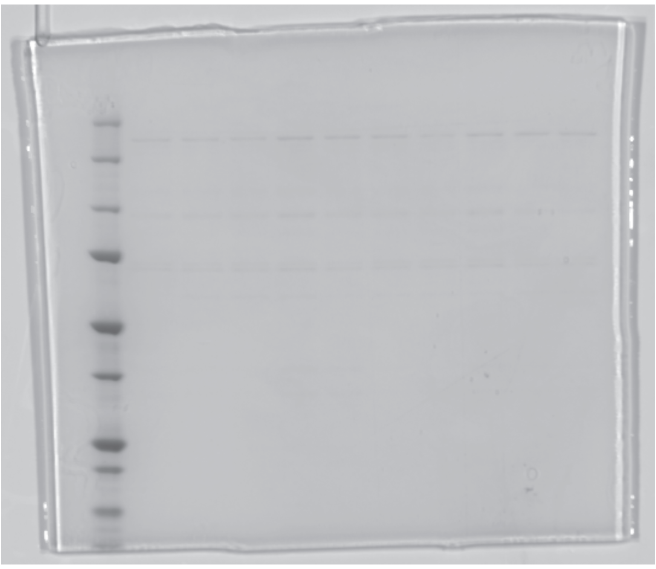

Fig S2H

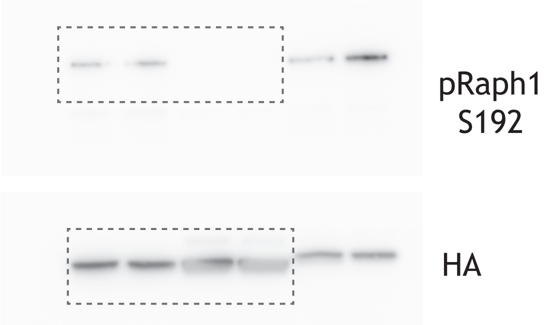

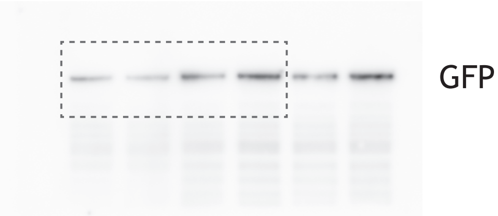

Fig S3A

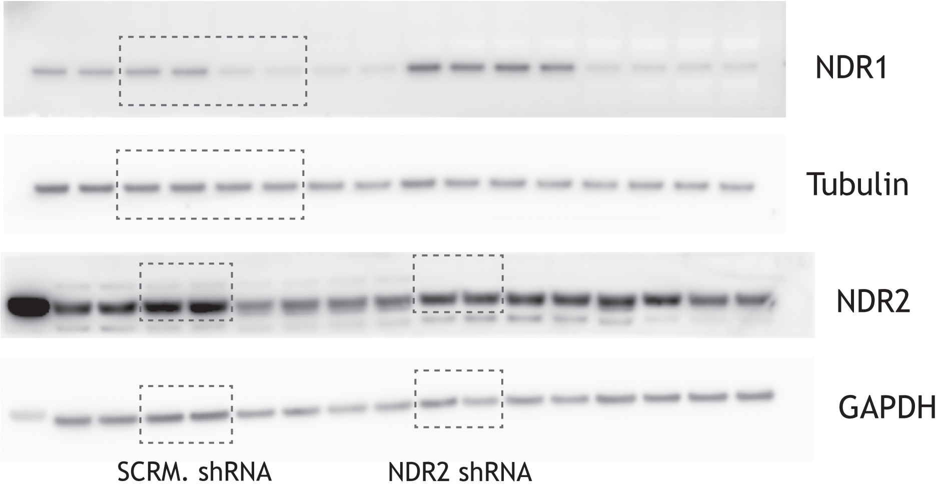

Fig S3B

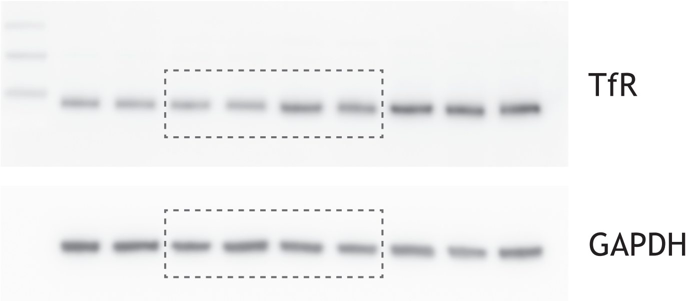

Fig S3D

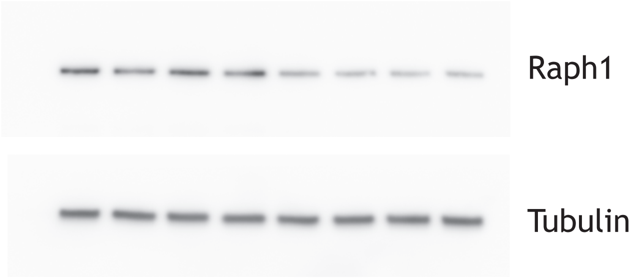

Fig S4A

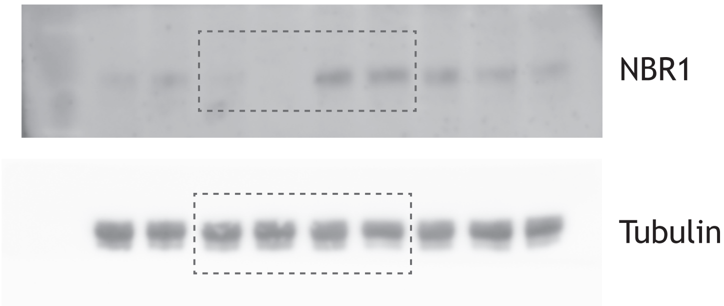

Fig S4D

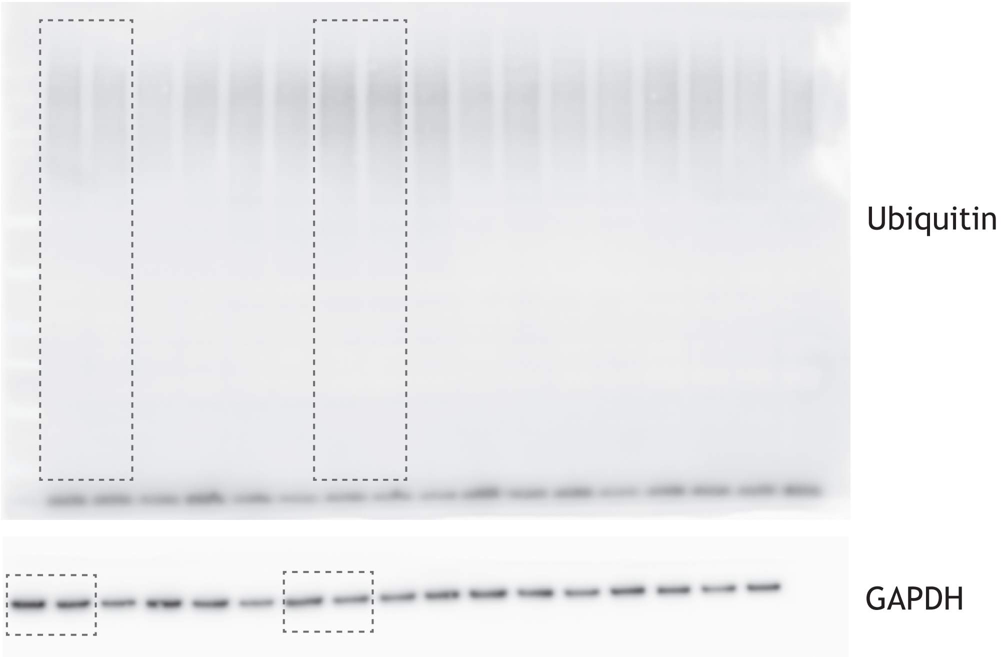

Fig S4G

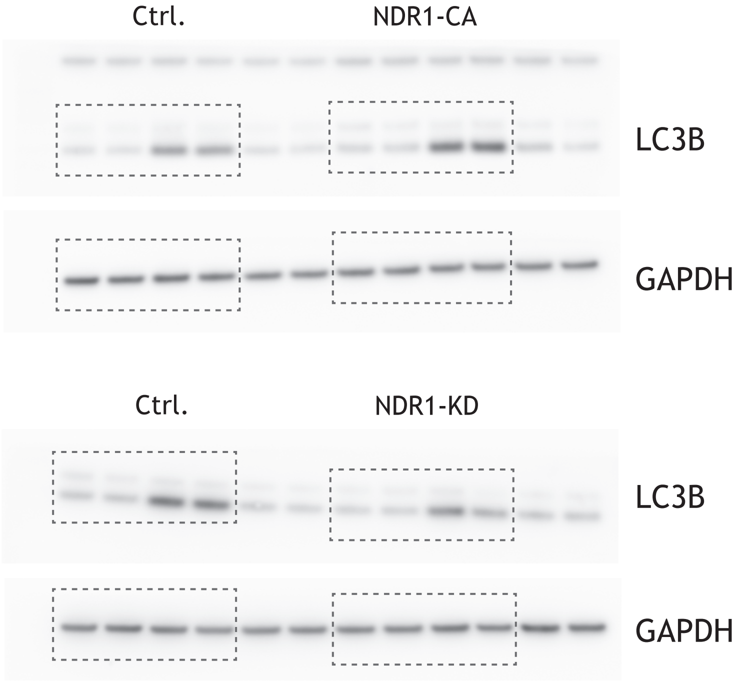

Fig S1B

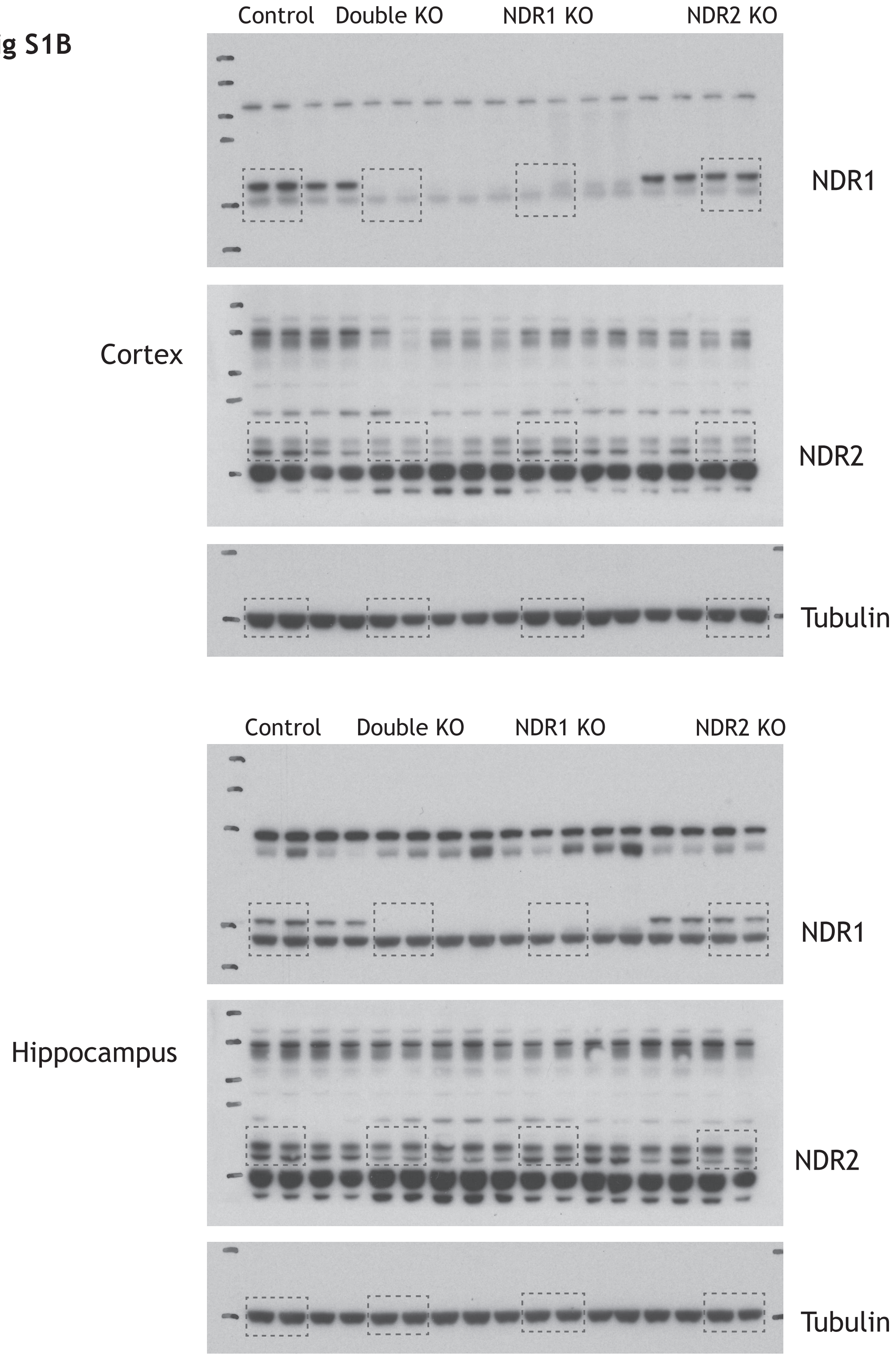
Control
Double KO
NDR1 KO
NDR2 KO
Cortex
NDR1
NDR2
Tubulin
Control
Double KO
NDR1 KO
NDR2 KO
Hippocampus
NDR1
NDR2
Tubulin
