# Supplementary material for: Loss of NDR1/2 kinases impairs endomembrane trafficking and autophagy leading to neurodegeneration
Source: Life Sci Alliance. 2022 Nov 29;6(2):e202201712. doi: 10.26508/lsa.202201712 (PMC9711861; doi:10.26508/lsa.202201712)
Supplement: Supplementary file 12 [file LSA-2022-01712_Supplemental_Data_1.docx]

**Packages used:**

**ProteoViz:** [**https://github.com/ByrumLab/ProteoViz.git**](https://github.com/ByrumLab/ProteoViz.git)

**Proteus:** [**https://github.com/bartongroup/Proteus.git**](https://github.com/bartongroup/Proteus.git)

**Legend:**

| Script 1 – Make Metadata | |
| --- | --- |
| Purpose | Files used |
| Creates metadata by extracting necessary data columns. | proteinGroups.txt (MaxQuant)  Phospho (STY)Sites.txt (MaxQuant) |
| Script 2 – Total Proteome Analysis | |
| Purpose | Files used |
| Data filtering of total proteome   - Reverse - Potential contaminant - Only identified by site   Data normalisation of total proteome using CONSTANd (Proteus)  Statistical comparison using limma | proteinGroups.txt (MaxQuant)  Sample_metadata.tsv (User-defined)  Contrast_matrix.tsv (User-defined)  Protein_metadata.tsv (ProteoViz-Script1) |
| Script 3 – Global Phosphoproteome Analysis | |
| Purpose | Files used |
| Data filtering of phosphoproteome   - Reverse - Potential contaminant   Data normalisation of phosphoproteome using CONSTANd (Proteus)  Statistical comparison using limma  Consensus motif column generation | Phospho (STY)Sites.txt (MaxQuant)  Sample_metadata.tsv (User-defined)  Contrast_matrix.tsv (User-defined)  Phospho_metadata.tsv (ProteoViz-Script1) |

**Sample_metadata.tsv:**

| Sample | Enrichment | Batch | Replicate | Pool | Sample_name | Model_group |
| --- | --- | --- | --- | --- | --- | --- |
| Reporter_intensity_corrected_1 | Lysate | 0 | 1 |  | Ctr 1 | Ctr |
| Reporter_intensity_corrected_2 | Lysate | 0 | 2 |  | Ctr 2 | Ctr |
| Reporter_intensity_corrected_3 | Lysate | 0 | 3 |  | Ctr 3 | Ctr |
| Reporter_intensity_corrected_4 | Lysate | 0 | 4 |  | Ctr 4 | Ctr |
| Reporter_intensity_corrected_5 | Lysate | 0 | 5 |  | Ctr 5 | Ctr |
| Reporter_intensity_corrected_6 | Lysate | 0 | 1 |  | dKO 1 | dKO |
| Reporter_intensity_corrected_7 | Lysate | 0 | 2 |  | dKO 2 | dKO |
| Reporter_intensity_corrected_8 | Lysate | 0 | 3 |  | dKO 3 | dKO |
| Reporter_intensity_corrected_9 | Lysate | 0 | 4 |  | dKO 4 | dKO |
| Reporter_intensity_corrected_10 | Lysate | 0 | 5 |  | dKO 5 | dKO |
| Reporter_intensity_corrected_1 | Phos | 0 | 1 |  | Ctr 1 | Ctr |
| Reporter_intensity_corrected_2 | Phos | 0 | 2 |  | Ctr 2 | Ctr |
| Reporter_intensity_corrected_3 | Phos | 0 | 3 |  | Ctr 3 | Ctr |
| Reporter_intensity_corrected_4 | Phos | 0 | 4 |  | Ctr 4 | Ctr |
| Reporter_intensity_corrected_5 | Phos | 0 | 5 |  | Ctr 5 | Ctr |
| Reporter_intensity_corrected_6 | Phos | 0 | 1 |  | dKO 1 | dKO |
| Reporter_intensity_corrected_7 | Phos | 0 | 2 |  | dKO 2 | dKO |
| Reporter_intensity_corrected_8 | Phos | 0 | 3 |  | dKO 3 | dKO |
| Reporter_intensity_corrected_9 | Phos | 0 | 4 |  | dKO 4 | dKO |
| Reporter_intensity_corrected_10 | Phos | 0 | 5 |  | dKO 5 | dKO |

**Contrast_matrix.tsv:**

| Contrast_name |
| --- |
| dKO - Ctr |

**Script 1 – Make Metadata**

library(tidyverse)

if(!dir.exists("data")){dir.create("data")}

df <- read_tsv("txt/proteinGroups.txt", guess_max = 10000) %>%

{set_names(., gsub(" ", "_", names(.)))}

df %>%

select(Majority_protein_IDs, Fasta_headers, Score, id) %>%

mutate(Description = str_extract(Fasta_headers, "(?<= )[^\\|]+(?= OS\\=)"),

Gene_name = str_extract(Fasta_headers, "(?<=GN\\=)[^\\|]+(?= PE\\=)"),

Uniprot_ID = str_extract(Fasta_headers, "(?<=\\|)[^\\|]+(?=\\|)")) %>%

write_tsv("data/Protein_metadata.tsv")

phos_df <- read_tsv("txt/Phospho (STY)Sites.txt", guess_max = 20000) %>%

{set_names(., gsub(" ", "_", names(.)))}

phos_df %>%

select(Proteins:Score, Amino_acid, Sequence_window, `Phospho_(STY)_Probabilities`, Charge, id:Evidence_IDs) %>%

mutate(Description = str_extract(Fasta_headers, "(?<= )[^\\|]+(?= OS\\=)"),

Gene_name = str_extract(Fasta_headers, "(?<=GN\\=)[^\\|]+(?= PE\\=)"),

Uniprot_ID = str_extract(Fasta_headers, "(?<=\\|)[^\\|]+(?=\\|)"),

Flanking = gsub("\\;.*$", "", Sequence_window) %>%

str_sub(9,23) %>%

paste0("-p")) %>%

write_tsv("data/Phospho_metadata.tsv")

**Script 2 – Total Proteome Analysis**

library(tidyverse)

library(limma)

df <- read_tsv("txt/proteinGroups.txt", guess_max = 20000) %>%

{set_names(., gsub(" ", "_", names(.)))}

sample_df <- read_tsv("Sample_metadata.tsv") %>%

mutate(Sample_name = factor(Sample_name, levels = unique(Sample_name)),

Model_group = factor(Model_group, levels = unique(Model_group))) %>%

#For protein

filter(grepl("Lysate", Enrichment))

contrast_df <- read_tsv("contrast_matrix.tsv")

df1 <- df %>%

filter(is.na(Reverse),

is.na(Potential_contaminant),

is.na(Only_identified_by_site))

df1[21:30][df1[21:30] == 0] <- NA

# using RAS (constrained standardisation CONSTANd) to normalise data

# the mean in each column and row equals to 1/n, where n is the number of columns and rows, respectively

# this bit of code was taken from Proteus package

RAS <- function(K, max.iter=50, eps=1e-5) {

n <- ncol(K)

m <- nrow(K)

# ignore rows with only NAs

good.rows <- which(rowSums(!is.na(K)) > 9)

K <- K[good.rows, ]

cnt <- 1

repeat {

row.mult <- 1 / (n * rowMeans(K, na.rm=TRUE))

K <- K * row.mult

err1 <- 0.5 * sum(abs(colMeans(K, na.rm=TRUE) - 1/n))

col.mult <- 1 / (n * colMeans(K, na.rm=TRUE))

K <- t(t(K) * col.mult)

err2 <- 0.5 * sum(abs(rowMeans(K, na.rm=TRUE) - 1/n))

cnt <- cnt + 1

if(cnt > max.iter || (err1 < eps && err2 < eps)) break

}

# reconstruct full table

KF <- matrix(NA, nrow=m, ncol=n)

KF[good.rows, ] <- K

return(KF)

}

normalizeTMT <- function(datf, max.iter=50, eps=1e-5) {{

datf[21:30] <- RAS(datf[21:30])

}

return(datf)

}

df1n <- normalizeTMT(df1)

df2 <- df1n %>%

mutate_at(vars(matches("corrected.*")), log2)

df2a <- df2 %>%

select(id, one_of(sample_df$Sample)) %>%

gather(Sample, Intensity, one_of(sample_df$Sample))

df2b <- df2a %>%

right_join(sample_df) %>%

group_by(id, Batch) %>%

group_by(Sample) %>%

ungroup()

df2b %>%

select(-Sample) %>%

write_tsv("data/Normalized_proteingroup_intensities.tsv")

# Limma -------------------------------------------------------------------

# Make model matrix and contrasts

model_df <- sample_df %>%

filter(!is.na(Sample_name))

design <- model.matrix(~ 0 + model_df$Model_group)

colnames(design) <- unique(model_df$Model_group)

cont_table <- makeContrasts(contrasts = as.list(contrast_df$Contrast_name), levels = model_df$Model_group)

Comparisons <- dimnames(cont_table)$Contrasts

# Format for Limma

df3 <- df2b %>%

filter(!is.na(Sample_name)) %>%

select(id, Sample_name, Intensity) %>%

spread(Sample_name, Intensity) %>%

as.data.frame() %>%

write_tsv("data/Protein_limma_input.tsv") %>%

column_to_rownames("id")

fit <- lmFit(df3, design)

cont_fit <- contrasts.fit(fit, cont_table)

fit2 <- eBayes(cont_fit)

#Extract topTable for each contrast

f1 <- function(x1){

a1_name <- colnames(fit2$coefficients)[[x1]]

a1 <- topTable(fit2, x1, number = Inf) %>%

rownames_to_column("id") %>%

mutate(Comparison = a1_name) %>%

mutate(id = as.integer(id)) %>%

as_tibble() %>%

left_join(df %>% select(id),

by = "id")

a1

}

df4 <- map_df(seq_along(Comparisons), f1) %>%

filter(!is.na(adj.P.Val))

df4 %>%

write_tsv("data/Protein_limma_output.tsv")

spread_protein_limma <- df4 %>%

select(id, logFC, adj.P.Val, P.Value, Comparison) %>%

gather(Type, Value, logFC, adj.P.Val, P.Value) %>%

unite(Type, Comparison, Type, sep = " ") %>%

spread(Type, Value) %>%

mutate(id = as.integer(id))

protein_meta <- read_tsv("data/Protein_metadata.tsv")

protein_quantitative <- df3 %>%

rownames_to_column("id") %>%

mutate(id = as.integer(id))

#For filtering, only include proteins identified in at least 1 sample

samples <- as.character(na.omit(sample_df$Sample_name))

summarized_protein <- protein_meta %>%

left_join(protein_quantitative) %>%

left_join(spread_protein_limma) %>%

filter_at(vars(one_of(samples)), any_vars(!is.na(.))) %>%

write_tsv("data/Protein_summarized_data.tsv")

**Script 3 – Global Phosphoproteome Analysis**

library(tidyverse)

library(limma)

#Run Protein_Limma.R first.

df <- read_tsv("txt/Phospho (STY)Sites.txt", guess_max = 20000) %>%

{set_names(., gsub(" ", "_", names(.)))} %>%

filter(

is.na(Reverse),

is.na(Potential_contaminant)

)

df0 <- read_tsv("txt/Phospho (STY)Sites.txt", guess_max = 20000)

sample_df <- read_tsv("Sample_metadata.tsv") %>%

mutate(Sample_name = factor(Sample_name, levels = unique(Sample_name)),

Model_group = factor(Model_group, levels = unique(Model_group))) %>%

#For protein

filter(grepl("Phos", Enrichment))

contrast_df <- read_tsv("contrast_matrix.tsv")

#Filters for class I sites, selects relevant columns, separates by phosphosite number

df1 <- df %>%

select(id, Protein_group_IDs, matches("corrected.*\\_{3}[[:digit:]]$")) %>%

gather(Sample, Intensity, everything(), -id, -Protein_group_IDs) %>%

separate(Sample, into = c("Sample", "Phos_number"), sep = "___") %>%

right_join(sample_df)

#Normalizes to pool, then centers distribution around median = 0, then normalizes to protein fold change

df2 <- df1 %>%

filter(Intensity > 0)

# Limma -------------------------------------------------------------------

model_df <- sample_df %>%

filter(!is.na(Sample_name))

design <- model.matrix(~ 0 + model_df$Model_group)

colnames(design) <- unique(model_df$Model_group)

cont_table <- makeContrasts(contrasts = as.list(contrast_df$Contrast_name), levels = model_df$Model_group)

#Format for Limma

df3 <- df2 %>%

filter(!is.na(Sample_name)) %>%

mutate(Intensity = ifelse(is.nan(Intensity), NA_real_, Intensity)) %>%

unite(id_phos, id, Phos_number) %>%

select(id_phos, Sample_name, Intensity) %>%

spread(Sample_name, Intensity, fill = NA) %>%

write_tsv("data/Phospho_limma_input.tsv") %>%

as.data.frame() %>%

column_to_rownames("id_phos")

RAS <- function(K, max.iter=50, eps=1e-5) {

n <- ncol(K)

m <- nrow(K)

# ignore rows with only NAs

good.rows <- which(rowSums(!is.na(K)) > 9)

K <- K[good.rows, ]

cnt <- 1

repeat {

row.mult <- 1 / (n * rowMeans(K, na.rm=TRUE))

K <- K * row.mult

err1 <- 0.5 * sum(abs(colMeans(K, na.rm=TRUE) - 1/n))

col.mult <- 1 / (n * colMeans(K, na.rm=TRUE))

K <- t(t(K) * col.mult)

err2 <- 0.5 * sum(abs(rowMeans(K, na.rm=TRUE) - 1/n))

cnt <- cnt + 1

if(cnt > max.iter || (err1 < eps && err2 < eps)) break

}

# reconstruct full table

KF <- matrix(NA, nrow=m, ncol=n)

KF[good.rows, ] <- K

return(KF)

}

normalizeTMT <- function(datf1, max.iter=50, eps=1e-5) {{

datf1[1:10] <- RAS(datf1[1:10])

}

return(datf1)

}

df4 <- normalizeTMT(df3)

df4[, 1:10] <- log(df4[1:10], 2)

fit <- lmFit(df4, design)

Comparisons <- dimnames(cont_table)$Contrasts

cont_fit <- contrasts.fit(fit, cont_table)

fit2 <- eBayes(cont_fit)

ncol(fit2$contrasts)

#Extract topTable for each contrast

f1 <- function(x1){

a1_name <- colnames(fit2$coefficients)[[x1]]

a1 <- topTable(fit2, x1, number = Inf) %>%

rownames_to_column("id") %>%

separate(id, into = c("id", "Phos_number")) %>%

mutate(Comparison = a1_name) %>%

mutate(id = as.integer(id)) %>%

as_tibble() %>%

left_join(df %>% select(id),

by = "id")

a1

}

df5 <- map_df(seq_along(Comparisons), f1) %>%

filter(!is.na(adj.P.Val))

df5 %>%

write_tsv("data/Phospho_limma_output.tsv")

spread_phospho_limma <- df5 %>%

unite(id, id, Phos_number) %>%

select(id, logFC, adj.P.Val, Comparison, P.Value) %>%

gather(Type, Value, logFC, adj.P.Val, P.Value) %>%

unite(Type, Comparison, Type, sep = " ") %>%

spread(Type, Value) %>%

separate(id, into = c("id", "Phos_number"), sep = "_") %>%

mutate(id = as.integer(id))

phospho_meta <- read_tsv("data/Phospho_metadata.tsv")

phos_quantitative <- df4 %>%

rownames_to_column("id") %>%

separate(id, into = c("id", "Phos_number"), sep = "_") %>%

mutate(id = as.integer(id))

phospho_meta %>%

left_join(phos_quantitative) %>%

left_join(spread_phospho_limma) %>%

filter(!is.na(Phos_number)) %>%

write_tsv("data/Phospho_summarized_data.tsv")

phos <- read_tsv("data/Phospho_summarized_data.tsv")

NDR <- str_detect(phos$Flanking, "([A-Z])([A-Z])([H])([A-Z])([R])([A-Z])([A-Z])([TS])([A-Z])([A-Z])([A-Z])([A-Z])([A-Z])([A-Z])([A-Z])")

phos$motif <- NDR

phos$motif <- gsub("TRUE", "NDR", phos$motif)

phos %>% write_tsv("data/Phospho_summarized_data_motif.tsv")
